# Identifying challenges to manage body weight variation in pig farms implementing all-in-all-out management practices and their possible implications for animal health: a case study

**DOI:** 10.1186/s40813-021-00190-6

**Published:** 2021-01-11

**Authors:** Maria Rodrigues da Costa, Edgar García Manzanilla, Alessia Diana, Nienke van Staaveren, Alberto Torres-Pitarch, Laura Ann Boyle, Julia Adriana Calderón Díaz

**Affiliations:** 1 Pig Development Department, Teagasc Grassland Research and Innovation Centre, Moorepark, Fermoy, Co. Cork Ireland; 2 School of Veterinary Medicine, University College Dublin, Belfield, Dublin, Ireland; 3 Present Address: Epidemiology research unit, Scotland’s rural College (SRUC), IV2 5NA Inverness, Scotland, UK; 4 Present Address: Department of Agronomy, Food, Natural resources, Animals and Environment (DAFNAE), University of Padova, Viale dell’Università 16, 35020 Legnaro, PD Italy; 5 Present Address: Department of Animal Biosciences, University of Guelph, Guelph, Ontario N1G 2W1 Canada; 6 School of Agriculture and Food Science, University College Dublin, Belfield, Dublin, Ireland; 7 Present Address: Trouw Nutrition España, Tres Cantos, Madrid, Spain

**Keywords:** Animal movements, Delayed pigs, Early weaning, Pig sorting, Production flow, Swine

## Abstract

**Background:**

Managing body weight (BW) variation is a challenge in farrow-to-finish farms implementing all-in/all-out (AIAO) production systems due to the lack of “off-site” facilities to segregate slow growing pigs (SGP). This case study investigated different approaches to managing BW variation in a farrow-to-finish commercial pig farm with a self-declared AIAO management and the possible implications for animal health.

**Case presentation:**

A total of 1096 pigs (1047 pigs born within 1 week plus 49 pigs born 1 week later) were tracked until slaughter as they moved through the production stages. Piglets were individually tagged at birth and their location on the farm was recorded on a weekly basis. In total, 10.3% of pigs died during lactation. Four main cohorts of pigs were created at weaning and retrospectively identified: **cohort 1 =** pigs weaned at 21 days (4.5%); **cohort 2 =** pigs weaned at 28 days (81.0%), which was sub-divided at the end of the first nursery stage into ***sub****-****cohort 2a*** **=** pigs split at 3 weeks post-weaning (29.7%); ***sub-cohort 2b*** = pigs split at 3 weeks post-weaning from cohort 2a and split again 5 weeks post-weaning (35.5%) and ***sub-cohort 2c*** = remaining smaller size pigs from cohort 2b (10.9%); **cohort 3 =** pigs weaned at 35 days (2.7%) and **cohort 4** = pigs weaned at 49 days (1.5%) that were later mixed with SPG, delayed pigs from other cohorts and sick/injured pigs that recovered. Four strategies to manage BW variation were identified: i) earlier weaning (cohort 1); ii) delayed weaning of SGP (cohort 3 and 4); iii) re-grading pens by BW (sub-cohorts 2a, 2b and 2c) and, iv) delayed movement of SGP to the next production stage (several pigs from all cohorts). A higher percentage of delayed pigs presented pericarditis, pleurisy and enzootic pneumonia like lesions at slaughter compared with pigs under other strategies.

**Conclusion:**

A variety of management practices were implemented to minimise BW variation during the production cycle. However, several cohorts of pigs were created disrupting AIAO management. Earlier weaning should only be practiced under specific circumstances where optimal animal health and welfare are guaranteed. Delayed weaning of SGP and delaying pigs to move to the next production stage could negatively affect animal health and should be avoided.

## Background

All-In/All-Out (AIAO) production systems improve growth performance and feed efficiency and reduce the risk of disease transmission [1, 2]. In such systems, animals are grouped together based on age. Pigs are transferred together into new accommodation as they move through the production stages with enough time allowed between batches to clean, disinfect, and dry the facilities. However, natural variation in growth performance exists between pigs from the same batch with up to 11% of pigs considered as slow growers [3–5]. Slow growth could be due to reduced growth potential [6], light birth and/or weaning weight [5–7] or presence of disease [8]. Slow growing pigs usually need longer time in either the nursery or finisher stages to reach slaughter weight which is associated with a greater risk of disease and economic losses [4, 9]. Indeed, a disadvantage of AIAO systems is the growth variation which is associated with inefficient pen utilisation [10]. It is recommended that slow growing and *pull out* (i.e. sick pigs that are segregated from the “normal” production flow) pigs should be moved to an “off-site” facility and mixing of different age groups should be avoided. In practice, adhering to such recommendations poses management challenges in most farms where animals are kept on the same site for the entire production period and producers try to maximise space usage. Although a high proportion of farmers claim to implement AIAO [11], re-grading pens by body weight (BW) on transfer to the next production stage [4] and mixing different age groups are common practices used to manage BW variation. It is likely that farmers, perhaps inadvertently, implement a repertoire of practices to manage BW variation within a batch of pigs throughout the production cycle. This is done in an effort to send more uniform batches of pigs to slaughter and to adhere to abattoir guidelines avoiding financial penalties [12]. However, such practices disrupt AIAO production and resemble more a continuous animal flow management system affecting animal performance, health and welfare indicators [4, 11, 13]. The objective of this case study was to identify different management approaches to control BW variation and their possible implications for animal health in a farrow-to-finish pig commercial farm with a self-declared AIAO management policy.

## Case presentation

The information presented in this case study is part of a larger project investigating respiratory disease on Irish pig farms, associated risk factors, and the relationship with performance, welfare and antimicrobial use. This was an observational study, whereby pigs were managed as per routine farm practice. Originally, 1047 pigs born within 1 week were individually tagged at birth. Information on birth weight, BW at 28 days of age, number of piglets born alive per litter, dam parity, cold carcass weight and presence of pleurisy, enzootic pneumonia (EP)-like lesions and pericarditis at slaughter were recorded for each pig. An additional group of 49 pigs born the following week were tagged at weaning as they were weaned with the original 1047 tagged pigs. Litter of origin or slaughter records were not available for these 49 pigs. All pigs were managed as per usual practice on the farm and the weekly location of the tagged pigs was recorded.

Pigs originated from a 1500 sow farrow-to-finish commercial farm in Ireland positive for *Mycoplasma hyopneumoniae, Actinobacillus pleuropneumoniae* and swine influenza A virus. This farm declared that it followed an AIAO policy whereby pigs would spend 8 weeks in the nursery stage after weaning (divided in two sub-stages of 4 weeks each – first and second nursery stages), 4 weeks in the grower stage and 8 weeks in the finisher stage. In each stage, rooms and pens had the same design and environmental control. The nursery facilities (i.e. 11 rooms with 16 pens each with fully slatted plastic floors) and grower facilities (i.e. 7 rooms with 16 pens each with fully slatted concrete floors) had an automatic temperature control system with fans in the ceiling while finisher facilities (38 individual trowbridge houses with fully slatted concrete floors) had natural ventilation. In each stage, pens were equipped with a wet/dry probe feeder with eight available spaces. Pigs were wet-fed ad libitum a common nursery diet with 18.3% of crude protein (CP) and 10.5 M Joules of digestible energy (MJ/DE) per kg of feed; a grower diet with 18.1% CP and 10.0 MJ/DE per kg of feed, and a finisher diet with 16.9% CP and 9.9 MJ/DE per kg of feed. Pigs had ad libitum access to water via nipple drinkers in each pen.

A total of 194 (18%) pigs from the 1047 originally tagged pigs died during the study. Of those, 105 pigs (54.1% out of 194) died during the lactation period, 24 pigs (12.4% out of 194) died during the nursery stage, 4 pigs (2.1% out of 194) died during growing stage and 14 pigs (7.2% out of 194) died during the finishing stages. Finally, 47 pigs (24.2% out of 194) were euthanized due to the presence of abnormalities such as external lesions, hernias, tail loss, severe lameness, external abscesses and emaciation. Furthermore, 26 females (2.4% out of 1096) were selected as replacement gilts and removed from the study. Only 1 pig died from the 49 pigs tagged at weaning.

### Animal management

**Fig. 1:**
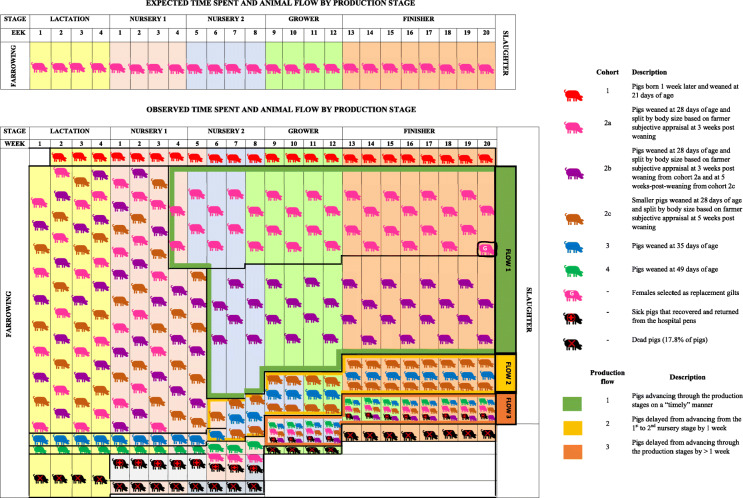
Expected versus observed time spent and animal flow by each production stage in a farrow-to-finish commercial pig farm where 1096 pigs were followed from birth to slaughter to investigate management approaches for body weight variation and their possible implications for animal health

Contrary to the purported AIAO policy followed in the farm, four different cohorts of pigs and three major production flows were identified according to the time pigs spent in each of the production stages (Fig. 1). The different cohorts were created at weaning (cohorts 1, 2, 3, 4). Additionally, cohort 2 was sub-divided into three sub-cohorts (sub-cohorts 2a, 2b, and 2c) during the first nursery stage. Descriptive statistics (mean ± SD) for litter of origin traits for the cohorts of pigs are presented in Table 1.

**Table 1:** Descriptive statistics (mean ± SD) for dam parity, number of piglets born alive per litter (BA), birth body weight (BW) and BW at 28 days of age of four main cohorts of pigs retrospectively identified according to the time they spent in each of the production stages in a study investigating management approaches for BW variation in a farrow-to-finish pig commercial farm and their possible implications for animal health

Cohort^**a**^	Sub-cohort^**b**^	Initial No. of pigs	Description	Dam parity		BA		Birth BW		BW at 28 d	
				Mean	SD	Mean	SD	Mean	SD	Mean	SD
1	–	49	Pigs born one week later and weaned at 21 d of age	NA^c^	NA	NA	NA	NA	NA	NA	NA
2	–	888	Pigs weaned at 28 d of age	3.3	1.44	13.1	2.53	1.4	0.30	7.3	1.49
	2a	326	Pigs weaned at 28 d of age. Split^d^ by size 3 weeks post-weaning from cohort 2	3.6	1.26	13.0	2.62	1.5	0.27	8.4	1.09
	2b	389	Pigs weaned at 28 d of age. Split^d^ by size 3 weeks post-weaning from sub-cohort 2a and 5 weeks-post weaning from sub-cohort 2c	3.2	1.51	13.0	2.59	1.4	0.30	7.1	1.18
	2c	120	Pigs weaned at 28 d of age. Smaller size pigs split^d^ by size 5 weeks post-weaning from sub-cohort 2b	3.2	1.47	13.3	2.25	1.3	0.29	5.5	0.94
3	–	30	Pigs weaned at 35 d of age	3.1	1.76	13.4	2.23	1.1	0.27	4.0	0.39
4		8	Pigs weaned at 49 d of age	3.0	1.41	13.5	4.41	1.1	0.38	3.3	0.86

^a^Cohorts formed at weaning

^b^Sub-cohorts formed during the first nursery stage

^c^*NA* No records available

^d^Split done based on farmer’s decisions and subjective appraisal

**Cohort 1,** was composed of 49 pigs (4.5% out of 1096 pigs) born 1 week later than the studied batch and weaned earlier than planned at 21 days of age. These pigs represented the fastest growing pigs of the batch they were born into, as per farmer subjective appraisal. Pigs in cohort 1 spent 5 weeks in the first nursery stage, 3 weeks in the second nursery stage, 4 weeks in the grower stage and 8 weeks in the finisher stage. Throughout the different production stages, these pigs were housed together in the same pens and only one of them died.

**Cohort 2** included 888 pigs (81% out of 1096 pigs) from the originally tagged batch weaned at 28 days as planned, moved to the first nursery stage facilities and re-organized in pens by size. A total of 38 pigs went to the hospital facilities during the first 3 weeks post-weaning. This cohort was sub-divided into three cohorts at the end of the first nursery stage. ***Sub-cohort 2a***: Three weeks post-weaning pens containing the heavier pigs (*n* = 326 pigs, 29.7% out of 1096 pigs) were sorted according to their BW/size (as per farmer subjective appraisal). *Smaller* pigs from each pen were removed, and the *heaviest* animals were moved into second nursery stage. Pigs in sub-cohort 2a continued to move through the different production stages in the same groups and spent 3 weeks in the second nursery stage, 5 weeks in the grower stage and 8 weeks in the finisher stage (Fig. 1). Twenty-one pigs from sub-cohort 2a died, 25 females were selected as replacement gilts at the end of the finisher period and 15 pigs were removed and delayed from advancing through the different production stages. ***Sub-cohort 2b:*** From the remaining 524 pigs in cohort 2 in the first nursery stage, 15 pigs died and at 5 weeks post-weaning the 509 remaining pigs were re-graded again by size (as per farmer subjective appraisal) and the *heaviest* of those pigs (*n* = 389 pigs, 35.5% out of 1096 pigs) were transferred to the second nursery stage. The *smaller* pigs were delayed once again in the first nursery stage accommodation. Pigs in sub-cohort 2b continued to move through the production stages in the same groups and spent 3 weeks in the second nursery stage, 4 weeks in the grower stage and 8 weeks in the finisher stage. Another 16 pigs in sub-cohort 2b died and 16 pigs were delayed from advancing through the different production stages. Pigs in sub-cohorts 2a and 2b followed a similar production flow (i.e. flow 1) and they were the fastest growing pigs from the batch. Information on number of pigs by production flow by cohort is presented in Table 2.

**Table 2:** Number and percentage of pigs dead, hospitalised, and selected as replacement gilt by production flow^a^ for four cohorts of pigs retrospectively identified according to the time they spent in each of the production stages in a study investigating management approaches for body weight variation in a farrow-to-finish pig commercial farm and their possible implications for animal health

Cohort^**b**^	Sub-cohort^**c**^	Initial No. of pigs	Description	Mortality		Hospital		Replacement gilts		Production flow					
										1		2		3	
				n	%	n	%	n	%	n	%	n	%	n	%
1	–	49	Pigs born one week later and weaned at 21 d of age	1	2.0	0	0.0	0	0	NA^d^	NA	NA	NA	NA	NA
2	–	888	Pigs weaned at 28 d of age	25	2.8	28	3.2	0	0.0	0	0.0	0	0.0	28	3.2
	2a	326	Pigs weaned at 28 d of age. Split^e^ by size 3 weeks post-weaning from cohort 2	21	6.4	0	0.0	26	7.7	265	81.3	0	0.0	15	4.6
	2b	389	Pigs weaned at 28 d of age. Split^e^ by size 3 weeks post-weaning from sub-cohort 2a and 5 weeks post-weaning from sub-cohort 2c	16	4.1	0	0.0	0	0.0	357	91.8	0	0.0	16	4.1
	2c	120	Pigs weaned at 28 d of age. Smaller size pigs split^e^ by size 5 weeks post-weaning from sub-cohort 2b	8	6.7	0	0.0	0	0.0	0	0.0	87	72.5	25	20.8
3	–	30	Pigs weaned at 35 d of age	2	6.7	0	0.0	0	0.0	0	0.0	25	83.3	3	10.0
4	–	8	Pigs weaned at 49 d of age	1	12.5	0	0.0	0	0.0	0	0.0	0	0.0	7	87.5

^a^Animals were slaughtered at 24 weeks of age and were retrospectively classified into three production flows (i.e. Flow 1 = normal, Flow 2 = delayed 1 week and Flow 3 = delayed > 1 week) according to the time they required to be moved to the next production stage

^b^Cohorts formed at weaning

^c^Sub-cohorts formed during the first nursery stage

^d^*NA* No records available

^e^Split done based on farmer’s decisions and subjective appraisal

***Sub-cohort 2c:*** At 7 weeks post-weaning, 120 pigs (10.9% out of 1096 pigs) were transferred to the second nursery stage, they continued to move through the production stages in the same groups and spent 4 weeks in the second nursery stage, 2 weeks in the grower stage and 8 weeks in the finisher stage (Fig. 1). From this sub-cohort, 8 pigs died, and 25 pigs were delayed from advancing through the different production stages.

Pigs in **cohort 3** (*n* = 30; 2.7% out of 1096 pigs) were weaned at 35 days of age; they spent 5 weeks in the first nursery stage, 4 weeks in the second nursery stage, 2 weeks in the grower stage and 8 weeks in the finisher stage (Fig. 1). Interestingly, pigs in cohort 3 were moved to the second nursery stage with pigs from cohort 2c and, from then on were managed as a single production flow (i.e. flow 2).

**Cohort 4:** Seventeen pigs (1.5% out of 1096 pigs) remained in the farrowing facilities. From these, seven pigs died and eight pigs were weaned at 49 days of age. These were the lightest pigs in the batch and spent 6 weeks in the first nursery stage, 3 weeks in the second nursery stage, 3 weeks in the grower stage and 5 weeks in the finisher stage (Fig. 1). However, starting from 5 weeks post-weaning until the end of the production cycle (i.e. 20 weeks post-weaning), these pigs were mixed with younger/similar sized pigs from the following batch, with delayed pigs from the other cohorts and with pigs that had returned from the hospital facilities (*n* = 28 pigs) having recovered from illness and/or injury. In fact, several of the latter pigs were subsequently delayed again and created a third production flow. This repeated delaying of pigs translated into some pigs spending extended periods in the different production stages. For instance, it took 8 weeks to move all pigs in this production flow to the second nursery stage, 6 weeks to move all pigs from the second nursery to the grower stage and 5 weeks to move all pigs from the grower to the finisher stage.

Based on the time spent in the different production stages by each cohort, four management approaches used to address BW variation were identified: i) earlier weaning (cohort 1), ii) delayed weaning of slow growing pigs (cohort 3 and cohort 4), iii) re-grading pens by BW (sub-cohorts 2a, 2b and 2c) and iv) delaying slow growing pigs (several pigs from all cohorts) from moving to the next production stage.

### Cold carcass weight and pluck lesions

Cold carcass weight was recorded by the slaughterhouse personnel and records were retrospectively acquired for pigs in cohort 2, cohort 3 and cohort 4. Pleurisy was scored using the Slaughterhouse Pleurisy Evaluation System (SPES [14];) and EP like lesions were scored according to the BPEX Pig Health Scheme [15] by a trained observer. However, due to the low numbers in the different scores, pleurisy and EP-like lesions were re-classified as present or absent. Additionally, presence or absence of pericarditis was recorded as per the decision of the acting veterinary inspector. Cold carcass weight (mean ± SD, kg) and number and percentage of pigs with presence of pleurisy, EP-like lesions and pericarditis at slaughter per cohort are presented in Table 3.

**Table 3:** Cold carcass weight (mean ± SD, kg) and number and percentage of pigs with presence of pleurisy, enzootic pneumonia (EP) like lesions and pericarditis at slaughter for four main cohorts of pigs retrospectively identified according to the time they spent in each of the production stages in a study investigating management approaches for body weight variation in a farrow-to-finish pig commercial farm and their possible implications for animal health

Cohort^**a**^	Sub-cohort^**b**^	Description	n	Cold carcass weight, kg		Pleurisy		EP		Pericarditis	
				Mean	SD	n	%	n	%	n	%
1	–	Pigs born one week later and weaned at 21 d of age	48	NA^c^	NA	NA	NA	NA	NA	NA	NA
2	2a	Pigs weaned at 28 d of age. Split^d^ by size 3 weeks post-weaning from group a group of 888 pigs	265	92.6	8.96	52	19.6	103	38.9	11	4.2
2	2b	Pigs weaned at 28 d of age. Split^d^ by size 3 weeks post-weaning from sub-cohort 2a and 5 weeks post-weaning from sub-cohort 2c	357	88.8	8.71	66	18.5	158	44.3	26	7.3
2	2c	Pigs weaned at 28 d of age. Smaller size pigs split^d^ by size 5 weeks post-weaning from sub-cohort 2b	87	86.6	9.36	21	24.1	36	41.4	10	11.5
3	–	Pigs weaned at 35 d of age	25	83.6	11.20	5	20.0	15	60.0	7	28.0
4	–	Pigs weaned at 49 d of age plus delayed pigs from the other cohorts and pigs that had returned from the hospital facilities	94	78.1	11.41	40	42.6	43	45.7	22	23.4

^a^Cohorts formed at weaning

^b^Sub-cohorts formed during the first nursery stage

^c^*NA* No records available

^d^Split done based on farmer’s decisions and subjective appraisal

### Sales income and margin over feed costs

It was not possible to accurately estimate production costs for the different cohorts as we lacked data on factors such as individual feed intake, veterinary treatments, labour, dead animal disposal, cost of transportation to the abattoir, among others. However, in order to illustrate the economic differences between the different cohorts, we estimated sales income by multiplying cold carcass weight by price per kg of meat produced and margin over feed costs as income per kg of meat minus feed cost per kg of meat for each cohort. Calculations were done considering a sales price of €1.79 per kg of meat and a feed cost of €1.08 per kg of meat which were the average prices in Ireland from November 2019 to October 2020 as per the Teagasc Pig e-Profit monitor. Results are presented in Table 4.

**Table 4:** Feed cost per meat produced^a^, sale income^b^ and margin over feed costs^c^ (mean ± SD, €/pig) for four main cohorts of pigs retrospectively identified according to the time they spent in each of the production stages in a study investigating management approaches for body weight variation in a farrow-to-finish pig commercial farm and their possible implications for animal health

Cohort^**d**^	Sub-cohort^**e**^	Description	n	Feed costs, €		Sales income, €		Margin over feed costs, €	
				Mean	SD	Mean	SD	Mean	SD
1	–	Pigs born one week later and weaned at 21 d of age	48	NA^f^	NA	NA	NA	NA	NA
2	2a	Pigs weaned at 28 d of age. Split^g^ by size 3 weeks post-weaning from group a group of 888 pigs	265	100.0	9.68	165.8	16.04	65.7	6.36
2	2b	Pigs weaned at 28 d of age. Split^g^ by size 3 weeks post-weaning from sub-cohort 2a and 5 weeks post-weaning from sub-cohort 2c	357	95.9	9.41	159.0	15.59	63.0	6.18
2	2c	Pigs weaned at 28 d of age. Smaller size pigs split^g^ by size 5 weeks post-weaning from sub-cohort 2b	87	93.5	10.11	155.0	16.75	61.5	6.65
3	–	Pigs weaned at 35 d of age	25	90.3	12.10	149.6	20.05	59.4	7.95
4	–	Pigs weaned at 49 d of age plus delayed pigs from the other cohorts and pigs that had returned from the hospital facilities	94	84.3	12.32	139.8	20.42	55.5	8.10

^a^Calculated by multiplying kg of cold carcass weight by €1.08 which was the average feed cost per kg of meat in Ireland from November 2019 to October 2020

^b^Calculated by multiplying kg of cold carcass weight by €1.79 which was the average sales price per kg of meat in Ireland from November 2019 to October 2020

^c^Calculated as sales income minus feed cost per kg of meat

^d^Cohorts formed at weaning

^e^Sub-cohorts formed during the first nursery stage

^f^*NA* No records available

^g^Split done based on farmer’s decisions and subjective appraisal

## Discussion and conclusions

All-in-all-out production improves growth performance [1, 2] and it is a critical management strategy to prevent the circulation of diseases among pigs throughout the production stages [16]. This is of particular importance in farrow-to-finish pig herds with weekly farrowing batches, as the contact between pigs of different age groups is potentially higher and older pigs could act as a carrier of pathogens to susceptible younger animals [17]. The AIAO policy needs to be implemented on farm in a way that no pig is delayed from moving to the next production stage (i.e. all forward policy). However, in practice, implementing such a strict AIAO policy is challenging if farmers try to maximise space usage by maintaining homogeneous weight groups and they do not have an off-site or on-farm specific facility for slow growing and/or delayed pigs. Nonetheless, even when an on-farm facility exists to house slow growing pigs, it can easily become a disease pool if not managed properly. Pigs from different age groups and health status are mixed there sharing the same air space and sometimes moved back to normal production flow with healthy animals [18].

In the absence of a specific facility, farmers use other practices to manage growth variation in pigs such as delayed weaning, re-grading groups by BW and/or delaying slow growing pigs from moving to the next production stage to allow them to catch up and reach adequate slaughter weights. These were the main practices observed in this farm for the management of BW variation. However, such practices could have implications for animal performance and health. In the present study, delayed pigs had lower carcass weight but also a greater percentage of pigs presented health conditions such as pleurisy and pericarditis compared with pigs that moved in a timelier manner throughout the production stages.

### Earlier weaning

In Europe, the recommended minimum age at weaning is 28 days [19] although mean average weaning age is lower in many European countries as well as in other major pig producing countries such as USA [20]. Earlier weaning could have many benefits for a farm such as producing more litters and pigs per sow per year [21]. In practice, earlier weaning should refer to weaning pigs some days earlier with respect to the average weaning age already in place on a given farm. For example, in a farm where pigs are routinely weaned at an average age of 35 days, pigs weaned at 28 days of age are considered as earlier weaned pigs. Also, earlier weaned pigs are an exception and not the normal practice on the farm. However, earlier weaning should only be considered under specific circumstances [19], ideally not before 21 days of age, and only for pigs that have reached adequate weaning weights to minimise post-weaning weight depression [22]. Earlier weaning at 21 days in farms where the general weaning policy is at 28 days is useful in many cross-fostering schemes [23] to create room for piglets in excess. In this particular farm, earlier weaning was not used regularly except where litters were bigger than expected and suckling piglets were heavy enough (around 7 kg). In terms of AIAO this practice is not ideal but the risk of transferring disease in this case is likely minimal as most of these animals may be healthy and close enough in age to the following batch. In this case study, pigs weaned earlier were kept in the same pen until the end of the production cycle (i.e. no pig was delayed at any given point or went to the hospital pens) showing no major issue to manage them. Farmers wanting to implement this management strategy on a regular basis should have dedicated pens for earlier weaned pigs and avoid mixing them with pigs weaned at an older age to minimize risk of disease transmission. Also, such pens should be clearly identified to ensure that earlier weaned pigs are vaccinated at the appropriate age. Unfortunately, we did not have information on carcass traits for the earlier weaned pigs; however, they went through the different production stages in a similar manner to those pigs in sub-cohort 2b suggesting they had similar growth.

### Delayed weaning

Approximately 4.2% of pigs were delayed at weaning. These pigs were, on average, 300 g lighter at birth and 3.8 kg lighter at 28 days of age compared with pigs weaned on a timely manner (i.e. 28 days of age). An increase in weaning age is associated with better post-weaning performance [24, 25]. It seems that spending extra time with the sow benefited pigs in cohort 3 as they were able to catch up and ended up spending a similar amount of time in each production stage (i.e. similar production flow) and having similar cold carcass weight as those in sub-cohort 2c. However, delaying weaning up to 49 d of age did not improve performance as this group of pigs (cohort 4) continued to grow slower and kept being delayed throughout the successive stages. Indeed, Camp Montoro et al. [26] observed that pigs with a body weight < 3.7 kg at 28 days of age would require approximately 20 days more to reach target body weight (i.e. 110 kg) compared with pigs over that threshold. Nonetheless, Camp Montoro et al. [26] reported that such pigs had similar feed conversion ratio to their bigger counterparts without any delays or special attention [27].

The percentage of pigs delayed at weaning may seem low. However, if we consider that these animals can be potential carries of diseases and are delayed repeatedly, the risk of this practice may be too high for such a small benefit. In this case study, the percentage of pigs with presence of pleurisy, EP-like lesions and pericarditis was higher for delayed weaned pigs although we cannot deduce whether these outcomes were causative or explanatory. For instance, pigs in cohort 4 were repeatedly re-mixed with younger pigs and pigs that had spent time in the hospital and were returned to the grow-finisher facilities once recovered. Such animals pose enormous risk of being carriers of pathogens. It is also possible that pigs in cohort 4 were more susceptible to diseases due to their lower weights. Further research is necessary regarding the impact of delayed weaning of slow growing pig on animal health. Finally, delayed weaning could have a negative impact on the overall performance of the farm. Sows weaned at 28 days would produce an average of 2.4 litters per year; this would be reduced to 2.3 litters per sow per year and 2.1 litters per sow per year for sows weaned at 35 days and 49 days post-farrowing, respectively. In turn, this decreases the mean number of herd litters per sow per year and the mean herd number of pigs produced per sow per year. This highlights the importance of moving all pigs forward throughout the different stages of the production cycle.

### Re-grading groups by BW

None of the cohorts followed the AIAO timeline declared by the farmer in each production stage. Most pigs were weaned at 28 days of age but subsequently re-graded into three different groups according to their size. Re-grading pigs by BW/size is a common practice in pig farms whereby producers try to minimise BW variation at the time of slaughter as abattoirs prefer more uniform batches [12, 28]. The result is the inadvertent creation of several “production flows” increasing the likelihood of disease transmission between pigs of different age groups [29] with different immune status [30]. Also, re-grading and therefore re-grouping, is associated with stress in pigs [31] and does not reduce the within pen BW variation [32, 33]. Lighter pigs continue to be lighter by the time of slaughter [28, 32, 33] as they still receive the same feed and are under the same management practices as heavier pigs. This farm may be an extreme example because pigs were re-graded three times with some of the pigs being moved faster through the different production stages while others spent more time in each stage than what was expected. If moving pigs faster through the production stages, they may be transferred to housing facilities that are inappropriate for their needs, at least during the initial days [11]. On the contrary, spending more time in each production stage could lead to reduced production efficiency. For example, nursery feed is more expensive due to its higher nutrient concentration and feeding pigs with these diets for periods longer than required increases feed costs.

There are two instances where pens of pigs might need to be re-mixed: 1) on transfer from one production stage to the next and 2) at the point of slaughter. Re-grading pigs on transfer from one production stage to the next should only occur if the number of pigs per pen will change in the following stage due to the design of available facilities. This is often the case in older units or where buildings are renovated in different stages. However, mixing of groups should be minimised and pigs must remain in their *original* groups as much as possible. This would contribute to reduced stress associated with mixing aggression to establish a new dominance hierarchy and minimise the risk of welfare related lesions [11, 34]. Also, by avoiding the re-grading of pigs, pathogens spread is minimised inside the group [35]. Re-grading pigs at the point of slaughter should be by split-marketing where heavier pigs are sent to slaughter once they have reached target slaughter weight. By doing this, pigs do not progress too quickly through the production stages assuring that their age appropriate needs are met by the housing environment [11] and SGP are allowed to remain the extra time required to reach adequate slaughter weight only during the finishing stage. In practical terms, pens should not be split-marketed more than twice, with the entire pen being sold on the second time [2]. By applying this approach, pig producers can avoid discounts for heavy animals and increase mean net margin per pig produced [36].

Nonetheless, an all-forward policy could also have some disadvantages such as inefficient space utilisation in pens with a high proportion of slow growing pigs. Also, the longer occupation period of pens housing slow growing pigs during the finish stage would reduce the number of pigs produced per pig space available per year. Furthermore, slow growing pigs are reported to have increased feed intake due to the extra time they spend in the finisher stage [26] likely increasing production costs. Additionally, some slow growing pigs would not be able to reach an acceptable slaughter weight in spite of the extra allotted time in the finisher stage and they will have to be sold at a discount price (e.g. lower base price or lower margin due to less kg of meat sold) or humanely euthanized.

Determining the economic impact of slow growing pigs is not simple because of the various causes for their slower growth (e.g. low birth/weaning weight, nutritional deficiencies, diseases, etc.) and the many factors involved in economic estimations. However, it is likely that slow growing pigs end up costing more money than they would ever return and slow growing pigs opportunity cost must be considered when making management decisions. The economic impact of slow growing pigs to farm profitability would depend on the pigs’ ability to convert feed efficiently, available pig spaces on-farm and farrowing rhythm, financial penalties at the abattoir, fluctuations in feed and pork prices, among others. Deen et al. [37] reported that price per kg of dead weight can decrease up to 60% for slow growing pigs if they are too light at slaughter while Calderón Díaz et al. [4] estimated a loss in sales income of €6.7 per slow growing pig. In this case study, pigs in cohort 2c returned 6.5% less income per pig than pigs in cohort 2a. This difference increases to 15.7% less income per pig when comparing pigs in cohort 4 (which had a higher proportion of slow growing pigs) with pigs in cohort 2a. Future studies should record individually factors such as feed intake, veterinary treatment, meat quality and penalties at the abattoir to accurately estimate production costs, sales and net profit for slow growing pigs.

### Delaying pigs from moving to the next production stage

Delayed pigs accounted for approximately 11% of pigs that reached slaughter age; they had lower cold carcass weight and over 40% of pigs presented pleurisy and EP-like lesions at slaughter. Indeed, delaying pigs from the normal production flow is associated with health problems. Fitzgerald et al. [38] reported increased antibody levels for *Actinobacillus pleuropneumoniae* in delayed pigs at slaughter and Calderón Díaz [4] reported that delayed pigs were more likely to be lame prior to slaughter, to have their heart condemned and present pericarditis and pleurisy at slaughter compared with pigs that followed the normal production flow. This supports the theory that delaying pigs from advancing through the production stages is associated with the re-circulation of disease and/or a higher risk of exposure to pathogens. This is due to sharing air space or being housed in the same pen with pigs that had returned from the hospital facilities having recovered from illness and/or injury. Nonetheless, further research is needed to elucidate whether the greater risk of disease in delayed pigs are causative or explanatory.

Slow growing pigs should only be delayed from the normal production flow ‘off-site’ or in a designated on-farm room to house them. Additionally, hospitalised pigs that have recovered should also remain segregated from the rest of the pigs. Segregating these pigs provides the opportunity to provide more feeder space and/or specialised diets that could help to improve growth performance and to minimise the risk of disease. Unfortunately, such facilities are normally not available in modern pig farms.

In conclusion, natural variation in growth performance poses a challenge for the implementation of a strict AIAO policy in farrow-to-finish pig farms and several management practices were implemented in a single farm. Although practices such as earlier weaning can provide some benefits, it should be implemented only when pigs exhibit superior growth rates during the lactation period. Other practices such as delayed weaning of slow growing pigs, delaying pigs from moving to the next production stage and constant re-grading of pens could negatively affect animal health. In practical terms we suggest that re-grading of pens should be kept to a minimum and no pigs should be delayed from moving through the different stages of production on a timeline manner. We propose the implementation of an “all forward” policy as it could be more easily implemented in farrow-to-finish pig farms. Keeping records of animal movements and identifying rooms and pens by age group could help to avoid mixing of older with younger pigs. Finally, pigs like those in cohort 4, slow growing pigs and sick pigs should be individually tagged and housed in a separate pen (i.e., they should not be mixed with other batches). This reduces the circulation of disease and the risk of exposure to pathogens.

## Data Availability

The datasets used for the results presented in this case study are available from the corresponding author upon reasonable request.
